# Integration with the human genome of peptide sequences obtained by high-throughput mass spectrometry

**DOI:** 10.1186/gb-2004-6-1-r9

**Published:** 2004-12-10

**Authors:** Frank Desiere, Eric W Deutsch, Alexey I Nesvizhskii, Parag Mallick, Nichole L King, Jimmy K Eng, Alan Aderem, Rose Boyle, Erich Brunner, Samuel Donohoe, Nelson Fausto, Ernst Hafen, Lee Hood, Michael G Katze, Kathleen A Kennedy, Floyd Kregenow, Hookeun Lee, Biaoyang Lin, Dan Martin, Jeffrey A Ranish, David J Rawlings, Lawrence E Samelson, Yuzuru Shiio, Julian D Watts, Bernd Wollscheid, Michael E Wright, Wei Yan, Lihong Yang, Eugene C Yi, Hui Zhang, Ruedi Aebersold

**Affiliations:** 1 Nestlé Research Center, 1000 Lausanne 26, Switzerland; 2 Institute for Systems Biology, 1441 N 34th Street, Seattle, WA 98103, USA; 3 Institute of Zoology, University of Zürich, CH-8057 Zürich, Switzerland; 4 Department of Pathology, University of Washington, Seattle, WA 98195-7705, USA; 5 Department of Microbiology, School of Medicine, University of Washington, Seattle, WA 98195, USA; 6 Department of Pediatrics, University of Washington, Seattle, WA 98195, USA; 7 National Cancer Institute, 37 Convent Drive, Bethesda, MD 20892, USA; 8 North Shore Long Island Jewish Research Institute, 350 Community Drive, Manhasset, NY 11030, USA; 9 Institute of Biotechnology, Swiss Federal Institute of Technology, ETH Hönggerberg, HPT E 78, CH-8093 Zürich, Switzerland

## Abstract

A crucial aim upon the completion of the human genome is the verification and functional annotation of all predicted genes and their protein products. Here we describe the mapping of peptides derived from accurate interpretations of protein tandem mass spectrometry (MS) data to eukaryotic genomes and the generation of an expandable resource for integration of data from many diverse proteomics experiments. Furthermore, we demonstrate that peptide identifications obtained from high-throughput proteomics can be integrated on a large scale with the human genome. This resource could serve as an expandable repository for MS-derived proteome information.

## Abstract 2

Peptides derived from protein tandem mass spectrometry data have been mapped to the human genome sequence forming an expandable resource for the proteomic data.

## Background

The recent definition of the complete nucleotide sequence of the human genome [1,2] has motivated the full annotation of the sequence. The true promise of the human genome project, to become the foundation for medical and biological research benefiting human health and quality of life [3], can only be realized if the coding sequences are conclusively identified, intron/exon structures are accurately described and the potential protein products from each gene in different tissues and cellular states are determined. Current methods for gene-prediction provide useful information but are still limited [4]. It is not presently possible to predict all features of the genome from its sequence alone. Therefore, the value of the human genome sequence can be enhanced through the collection of different types of experimental data and its integration and validation in a genomic context [5].

Current use of expressed sequence tags (EST) and full coding DNA (cDNA) sequences is extremely helpful in achieving complete genome annotation [6-9]. However, these data are not sufficient to unequivocally predict which proteins (and with what covalent structure) are expressed in a given tissue. The complete characterization of all proteins across disease states, tissues and stages of development can now be addressed through experimental protein identifications generated by proteomic methods. Experiments carried out over the past years have illustrated that peptides resulting from proteolytic digests of complex protein mixtures can be identified in a high-throughput mode using a combination of liquid chromatography (LC) and tandem mass spectrometry (MS/MS) (LC-MS/MS) [10-15]. Peptides are thus useful as the currency of MS/MS-based protein identification [16]. By combining a large number of experiments sampling different cell and tissue types, the observed peptides can be mapped onto the genome covering a significant part of its chromosomes.

## Results and discussion

To begin annotating the human genome with protein-level information, we have built PeptideAtlas. The generally applicable procedure to annotate eukaryotic genomes with peptide sequences can be applied when datasets are acquired using different experimental protocols. In each case, sample proteins were first proteolytically cleaved into peptides using the enzyme trypsin. The resulting peptide mixture was then subjected to chromatographic separation by strong cation exchange and reverse-phase capillary chromatography. In addition, those experiments using the ICAT (isotope-coded affinity tag) reagent for quantification included an avidin affinity-purification step to select peptides containing biotinylated, stable-isotope-tagged cysteines [16]. The resulting peptide pools were then analyzed by electrospray ionization (ESI)-MS/MS. The database search program SEQUEST [17] was used to assign the resulting MS/MS spectra to a peptide sequence. The confidence of these peptide assignments was evaluated using PeptideProphet [18]. All of the experimental data products, including PeptideProphet probability scores, are loaded into SBEAMS - Proteomics, a proteomics analysis database built as a module under the Systems Biology Experiment Analysis Management System (SBEAMS) framework. All of the identifications above a certain probability threshold within a specific set of experiments are extracted from the main database tables into another set of tables containing the attributes of each distinct peptide.

**Figure 1:**
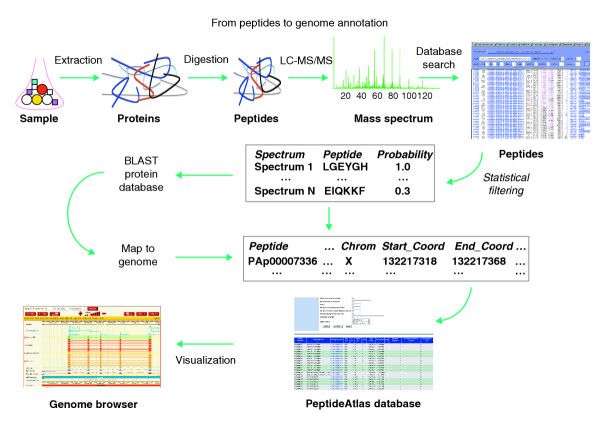
Analysis pipeline for the annotation of the human genome with high-quality peptide sequences derived from high-throughput MS analysis of biological samples.

**Figure 2:**
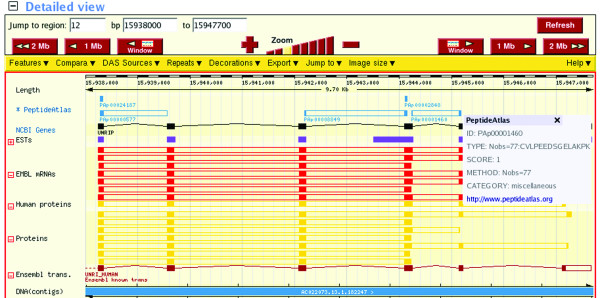
Visualization of PeptideAtlas peptide entries in the Ensembl DAS browser as a separate track at the top called PeptideAtlas, displayed as light blue rectangles. The Ensembl genome browser, here showing 10 kilobases (kb) on chromosome 12, can be used to zoom into the genome down to the nucleotide level. A light blue line connects peptides that map on intro/exon boundaries. Details about the peptide, including its unique identifier, peptide sequence, best PeptideProphet probability [22] (marked SCORE) and PeptideAtlas hyperlink are displayed.

Resulting from this, 26,840 distinct peptide sequences were identified from 224,973 spectra with identifications of a high probability (*P *≥ 0.9) of being correct. Each peptide is given a unique and stable identifier with an eight-digit number in the form PAp00000001. We then attempted to map the 26,840 distinct peptides to the human genome sequence using the analysis pipeline shown schematically in Figure 1. An example of visualizing the result in a genome browser is shown in Figure 2 (see Materials and methods for details). The mapping results are summarized in Table 1. The result of this process is stored as a freely available public resource, the human PeptideAtlas database [19].

The current build of PeptideAtlas contains peptide sequences identified in 52 proteomic experiments in which proteins were extracted from a particular cell or tissue type, digested with trypsin and analyzed with a mass spectrometer. The 52 proteomic experiments comprised 14 published as well as 38 unpublished human datasets from various cell types such as T cells, B cells, lymphocytes, lymphoblasts, hepatocytes, intestinal cells, hepatoma cells and others. The 14 published datasets contain 47% of the distinct peptides in PeptideAtlas. A full listing of all the experiments and samples currently in PeptideAtlas can be found at the project website [19]. The raw data for all published datasets is also provided in a repository there.

**Figure 3:**
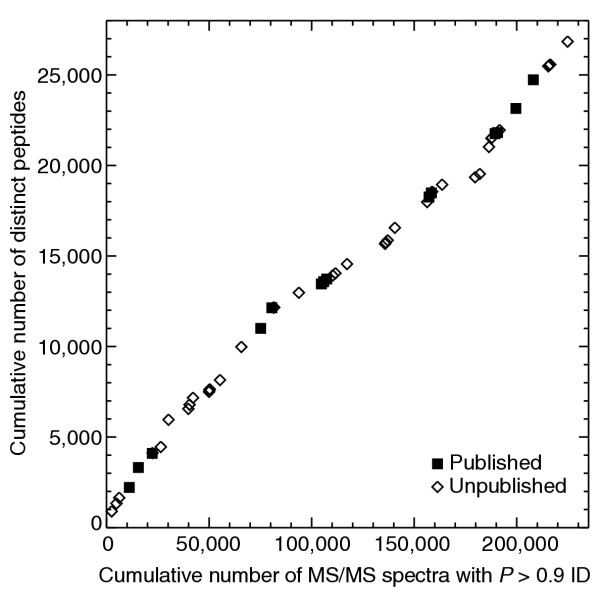
Cumulative number of distinct peptides as a function of the addition of more good spectra (identified with *P *≥ 0.9). Eventually the pattern is expected to show saturation, as most observable peptides will have been cataloged. However, at present there is no evidence of saturation and around 100 new peptides are still cataloged per 1,000 identified spectra added.

The cumulative number of distinct peptides as a function of the addition of identified spectra (with *P *≥ 0.9) in the atlas is shown in Figure 3. As most of the observable peptides in the proteome are matched with genes, the curve is expected to saturate and adding additional data will yield few new matched genes. However, the current behavior is still completely linear, with approximately 1 in 10 identified spectra contributing a previously uncataloged peptide. Each data point represents an added experiment; the experiments are presented approximately in chronological order of data collection. Among the 52 experiments, there is clearly great variability in the total number of identified spectra contributed as well as new distinct peptides contributed. A repeated, complex-sample experiment might yield many new spectra but few new distinct peptides, while a new sample of a type not previously analyzed might yield relatively few spectra but most of these might contribute a new distinct peptide.

Applying our pipeline described in Materials and methods, 25,754 of the 26,840 distinct peptides in PeptideAtlas were mapped to 9,747 (28.6 %) of the 34,091 human Ensembl proteins (version 22.34d.1, 2004-06-02). These proteins represent unique proteins or splice forms from 6,423 genes (27%) of human genes in Ensembl.

Some peptides have indistinguishable, perfect protein sequence matches to multiple proteins. These proteins are typically paralogs (protein families), protein isoforms or repeated protein domains in the human genome. We identified 3,718 proteins unambiguously by one or more 'discrete peptides' - peptides that map uniquely to a single protein - in the current build of PeptideAtlas. Those peptides are marked in the genome browser as 'discrete peptide'. 'Degenerate peptides' that map to several protein isoforms are also used to identify proteins. It would thus be more accurate to state that a product of a certain gene, rather than a certain protein, has been identified [20-22]. Moreover, the experimental data from those degenerate peptides generally do not allow differentiation between the sequence alternatives that exist in Ensembl. In fact, not all splicing variants that are in Swiss-Prot are also present in Ensembl and, therefore, it is impossible to ascertain the number of unambiguous identifications at the moment. This limitation underscores the requirement for mapping large-scale proteomic data to the human genome, such as presented in this report to aid in the generation of unambiguous sequence databases.

A significant number of distinct peptides (1,086), assigned by SEQUEST/ProteinProphet from over 5,000 MS/MS spectra, could not be mapped to Ensembl database version 22.34d.1. These peptides were identified by SEQUEST searches against the IPI database [23] or ABCC non-redundant protein database (NCI) [24]. These peptides are of special interest as they often document interesting biological phenomena such as single-nucleotide polymorphisms (SNPs) and novel splice variants, demonstrating the need for annotating the human genome sequence with high-quality experimental data obtained from expressed proteins. The existence of these sequences also illustrates the flux in the genome annotation and sequence databases. For example, in Ensembl version 18.34.1, only 92% of genes from the previous build were transferred across to the new build. The missing 8% were predominantly inappropriate protein-coding genes coming from large-scale cDNA projects, which have a number of artifactual errors, or from chimeric cDNA clones from cancer cell lines. Experimentally observed, unmapped peptides are an ideal source of information for refining genome assembly and gene prediction.

The absence of Ensembl matches does raise the question of whether these peptides are false positives or whether real proteins are missing in the Ensembl database. When these peptides were investigated in more detail it was found that nearly 100 were identified 10 or more times in several different experiments, and that many had protein sequence matches for Swiss-Prot entries. They are therefore likely to be true peptide attributions. For example, peptide PAp00000363 (AGKPVICATQMLESMIK) was identified 626 times at different charge states and with different mass modifications in 22 distinct experiments and mapped to KPY1_HUMAN, a pyruvate kinase M1 isozyme. Interestingly, the protein appears to have a likely SNP, which mutates the valine present in the Ensembl genome sequence to the isoleucine observed in PAp00000363.

**Figure 4:**
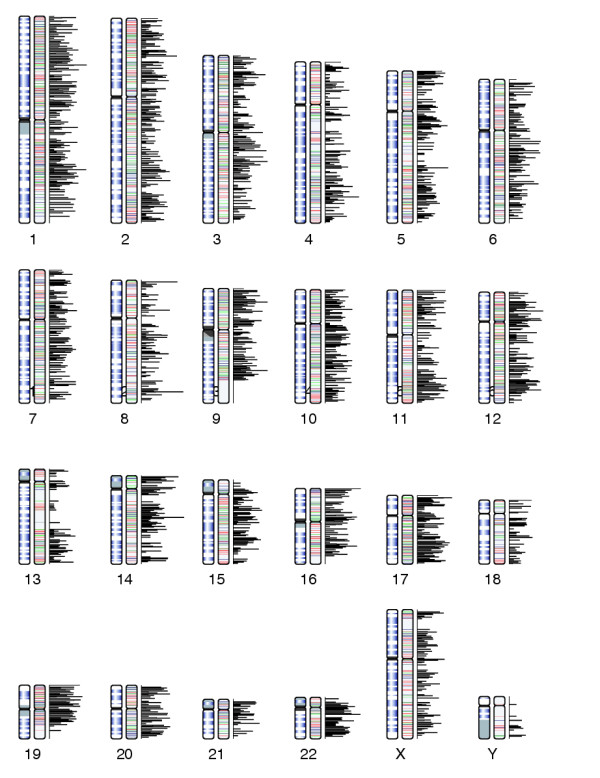
Distribution of PeptideAtlas peptides on the human genome. Each chromosome is described by three columns. The left-most column shows a chromosome's standard banding. The right-most column presents a histogram of the mapping of peptides to chromosomal regions; a line's length represents the number of peptides mapped to a chromosomal region. The central column indicates the over/under representation of peptides in a given region. Green regions represent more mapped peptides than expected at uniform random; red regions indicate fewer mapped peptides than expected at uniform random.

The 9,747 mapped proteins represent 28.6% of the predicted human proteome in Ensembl version 22.34d.1. The distribution of peptide matches to these proteins (Figure 4) revealed coverage of all chromosomes. Void areas were observed in the centromere region of chromosome 1 and the telomere regions of chromosomes 13, 14 and 15. These missing regions represent the unsequenced parts of human chromosomal heterochromatin structures and are therefore expected to be devoid of peptide matches. Very few peptides were observed mapped to chromosome Y.

The development of PeptideAtlas and a method for mapping observed peptides to the genome allows us to determine the distribution of multiple peptide hits to specific proteins and the distribution of peptide sequences that are present in multiple proteins. Also, in some cases splice junctions and gene boundaries could be confirmed. Our method allows us also to identify peptides corresponding to abundant proteins such as actin, elongation factor and glyceraldehyde-3-phosphate dehydrogenase, which are commonly identified in high-throughput LC-MS/MS experiments. These proteins are products of housekeeping genes, which are expressed most of the time in almost every tissue [25], or are structural proteins which are also known to be abundant in cells.

**Figure 5:**
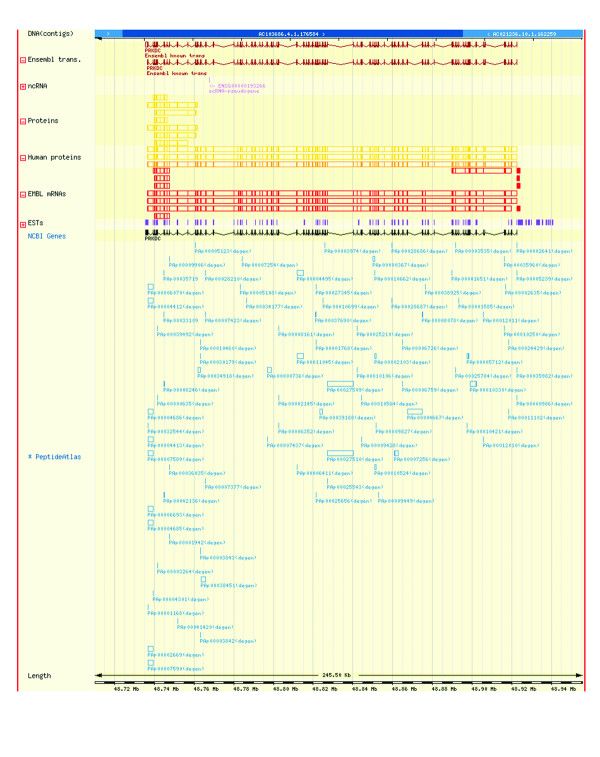
View of the DNA-dependent protein kinase catalytic subunit PRKDC gene (ENSG00000121031), which is matched by 90 distinct peptides in PeptideAtlas.

The identification of proteins that are specific to a given cell, tissue or disease state allows for the selection of marker proteins. The knowledge of a single marker, or a set of marker proteins, is crucial for the development of new strategies for rapid protein analysis and quantitative proteome profiling [16,26]. In PeptideAtlas we identify proteins to which two or more peptides map. In fact, for some proteins, 100 or more peptide matches were determined. These proteins were often unusually large in size and contained many exons. Examples of such proteins include the 1,462-amino-acid alpha-2-macroglobulin precursor (ENSP00000323929), which was matched by 161 peptides, or the 4,126-amino-acid DNA-dependent protein kinase catalytic subunit (ENSP00000313420) matched by 90 peptides (Figure 5), the 2,472-amino-acid spectrin alpha-chain protein (ENSP00000238302) with 102 peptides, and cytoplasmic 2 actin (ENSP00000331514) with 127 peptides.

**Figure 6:**
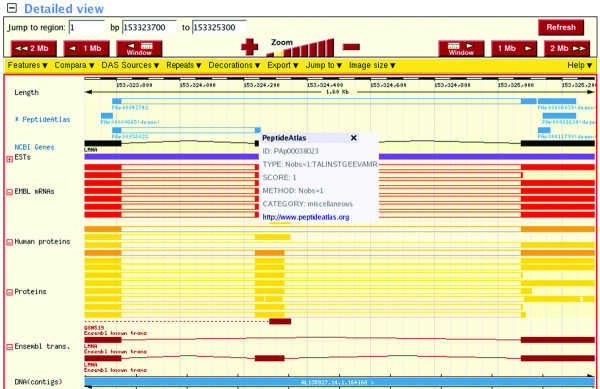
Example of peptides confirming a case of alternative splicing of the lamin A/C gene (LMNA). PAp00038023 was identified as part of protein ENSP00000310687 from the SiHa human cell line experiment. PAp00042742 was identified as part of protein ENSP00000292304 from a human B-cell experiment.

We also identified peptides whose amino-acid sequence is shared by members of protein families or shared domains among proteins in the genome. Peptides were matched to all identical sequences in all proteins. Multiple hits were possible and the resulting peptides were called degenerate peptides [22], in contrast to discrete peptides that matched one protein uniquely. For example, peptide PAp00001228 (CNGVLEGIR) matched to 26 proteins in the myosin family and peptide PAp00025728 (HCQLAIR) mapped to 23 proteins. Furthermore, our method was able to confirm intron/exon boundaries by identifying peptides that spanned these regions in a gene. We identified 4,800 intron/exon boundary-spanning peptides, corresponding to 2% of the splice junctions in the human Ensembl database, experimentally confirming specific intron/exon junctions. In most cases, these boundaries were already known to exist from cDNA information. However, using peptide information we were able to specifically confirm those boundaries on the level of expressed proteins. In one case (Figure 6) we observed a peptide confirming a skipped exon. This event was previously proposed to occur during expression of the A-type lamins in the lung adenocarcinoma cell line GLC-A1 [27]. The presence of some lamin A10 isoforms can easily be overlooked owing to their relatively low abundance. This new peptide information confirms the existence of this splice variant and shows that low-abundance proteins can be detected through the proteomics technologies described in this paper.

The need for public proteomics data repositories is recognized [28] and we intend PeptideAtlas to become a growing database and public resource. We have structured the system in a way that allows scientists to submit their own MS data for incorporation into PeptideAtlas, thus increasing the number of experiments and identified peptides. Naturally, to be useful for the project, inclusion of third-party data is dependent upon data compatibility and consistent data quality. Consequently, only data with accurate statistical measures of confidence computed by, for example, PeptideProphet, or another published and tested statistical algorithm, will be included. Datasets for which such statistical analyses have been performed can be submitted for incorporation following the procedure detailed at the PeptideAtlas website. Alternatively, data contributors can submit raw MS/MS data directly. This information should preferably be formatted into mzXML [29] or mzData (HUPO Proteomics Standards Initiative) which are open file formats for the representation of MS data. Other traditionally used data formats are accepted as well.

This data will then be searched by the PeptideAtlas curators using SEQUEST to correlate MS/MS spectra of peptides with amino-acid sequences using protein databases such as IPI, and the results will be further analyzed with PeptideProphet. An effort to add support for additional search engines is underway. This procedure will ensure the highest degree of consistency for the data in PeptideAtlas. In the future, the pipeline in general and the data submission process in particular, can be further improved and make compliant with the community accepted statistical data-validation standards and data file formats when such standards emerge [30]. Please see the submission section on the PeptideAtlas web-site for the most up-to-date submission methods and curator contact information. With an increasing number of included peptides, the utility of the resource will improve, as increasing numbers of genes, exons, transcripts and variant transcripts in many tissues and developmental stages will be verified on the protein level.

All MS/MS spectra are stored in the SBEAMS - Proteomics database, from which PeptideAtlas is derived. While at present it is not possible easily to access the MS/MS spectra starting from the public PeptideAtlas interface, this possibility could be added in the future. All spectra for published experiments are available in the mzXML files in the repository. Access to raw spectra can be beneficial for many applications not related to the main purpose of PeptideAtlas. Furthermore, because peptide modifications (for example, phosphorylation) are stored, this information could be displayed as well.

It is well understood and discussed in the literature [21] that all large-scale datasets obtained using high-throughput methods inherently contain a certain fraction of false-positive data. Thus, estimation of false-positive error rates is a very important but often challenging task. One significant advantage of the high-throughput pipeline implemented in this work is that computed peptide probabilities (here produced by PeptideProphet) allow estimation of the upper bound (most conservative estimate) of the false-positive identification error rates for any dataset submitted to PeptideAtlas. As the main purpose of PeptideAtlas is to map peptide identifications to the genome, the most relevant estimate of the false-positive error rates is the one at the level of distinct peptide assignments that have a defined mapping to Ensembl.

Initial datasets of peptide assignments to MS/MS spectra, obtained by searching acquired MS/MS spectra using the database search program SEQUEST, were statistically validated using the computational tool PeptideProphet. For each peptide assignment to an MS/MS spectrum, PeptideProphet computes a probability of its being correct, based on its database search scores, difference between the measured and theoretical peptide mass, the number of termini consistent with the type of enzymatic cleavage used, the number of missed cleavage sites and other factors. Probabilities computed by PeptideProphet have been shown to be accurate in the entire probability range and, therefore, can be used to compute the false-positive identification error rate (fraction of all identifications passing the filter that are incorrect) resulting from filtering each dataset using any minimum computed peptide probability threshold [18]. The false-positive identification error rates for the combined dataset of peptide assignments (all 52 experiments) filtered using minimum probability thresholds 0.7, 0.9, 0.95 and 0.99 are shown in Table 2.

To assess the effect of using a particular probability threshold on the number of peptides in the atlas, we ran the PeptideAtlas pipeline using probability thresholds *P *≥ 0.7, 0.9, 0.95 and 0.99. Decreasing the probability threshold increases the number of peptides, both correctly and incorrectly identified, and the corresponding proteins (Table 2). The most stringent threshold of *P* ≥ 0.99 produced 21,030 peptides with protein sequence matches (4,845 protein identifications), almost 8,400 fewer than the lowest threshold of 0.7 (2,252 fewer protein identifications). The *P* ≥ 0.9 threshold yielded 25,754 peptides with protein sequence matches at an estimated false-positive rate of less than 7%, and we selected this as an acceptable level for the default PeptideAtlas. The number of false-positive identifications could be reduced by selecting a higher threshold; however, a significant number of correct peptides and proteins would then also be eliminated. The additional peptides resulting from the low-probability threshold were valuable for adding additional peptide evidence in combination with higher-probability peptides corresponding to the same protein (peptides corresponding to proteins to which other peptides correspond are more likely to be correct than their probability value indicates [22]). We provide at our website the option for users to browse or download versions of the Atlas generated with the other *P *thresholds, which might be useful for some applications.

To validate our approach for general use in eukaryote genomes, we have extended our methods to peptides obtained from *Drosophila melanogaster *LC-MS/MS experiments. We collected data obtained from cytoplasmic, nuclear and membrane fractions derived from a *Drosophila *S2 Schneider cell line. The resulting 4,406 different peptides with *P* > 0.9 were compared to the 18,289 proteins (Ensembl fly database version 18.3a.1, 2003-07-01) using the same pipeline as described for human. From the fly, 3,107 proteins could be validated, representing 1,876 (14%) of the fly's genes. These results show that our method could easily be adapted to other organisms, thus opening up the way for comparative proteome-level evaluations of eukaryotic organisms.

## Conclusions

We have annotated the human genome with protein evidence for nearly 10,000 proteins. Although this number only represents a fraction of the genome and still contains some erroneous identifications, it is a first step towards the final goal: to fully annotate eukaryotic genomes via validation of expressed proteins. PeptideAtlas provides a method and a framework to accommodate proteome information generated by high-throughput proteomics technologies and is able to efficiently disseminate experimental data in the public domain. Its significance continues to grow as more data are submitted.

Moreover, PeptideAtlas also allows one to address the important question of how big the human proteome is. Due to the technical limitations of current proteomics technologies, it is not possible yet to determine the complete proteome in one experiment. However, if the data from diverse experiments, using different cellular compartments and enrichment methods were combined, the determination of the complete proteome could eventually be achieved. PeptideAtlas offers the framework to answer this question accurately and to determine the size of the complete human proteome using pooled experimental data. Furthermore, PeptideAtlas provides a resource for the development of new avenues of research. The dataset will provide a rich source of data for computational scientists to develop and test new algorithms for proteomic analysis, gene discovery and splice-variant prediction.

The methods described here, combined with the ever-increasing power of proteomics and bioinformatics technologies, will facilitate the determination or characterization of protein-coding genes, their features, and their processing and expression in relationship to the sequence of the human genome, thus contributing significantly to our understanding of genome structure.

## Materials and methods

### Pipeline

The assembly of experimentally derived distinct peptides is mapped to the human genome in the following way. First, we use BLAST [31] to match the peptides to the Ensembl human protein database. The Ensembl database project [32] provides a bioinformatics framework to organize biology around the sequences of large genomes and, furthermore, extensive resources and visualization options as well as remote access to the underlying relational databases [33]. The human genome sequence (release 22.34d.1, 2004-06-02) contains 23,758 genes and 34,091 gene transcripts. Second, complete matches, spanning each peptide's complete length, were used to determine human chromosomal coordinates. The method for retrieving chromosomal coordinates within the human genome accounts for splice junctions; in cases where a peptide maps onto a splice junction, it is projected to both parts of the chromosome, generating multiple sets of coordinates. Third, the results are loaded into a relational database. This database schema (available at the project website [19]) is able to accommodate data for different PeptideAtlas builds, for different organisms or different reference protein sequence sets as starting material and is thus extremely versatile. Fourth, visualization of the results was achieved using the Distributed Annotation System (DAS) (Figure 2) in conjunction with the Ensembl database. DAS allows sequence annotations to be decentralized among multiple third-party annotators and integrated on an as-needed basis by the Ensembl genome browser [34].

### Data collection

LC-MS/MS analysis was performed on LCQ, Ion-trap (Thermo Finnigan LCQ) and Q-Tof (Micromass Waters) instruments.

To estimate the false-positive error rate on the level of distinct peptide identifications, we first note that there is an almost 10-fold difference between the number of peptide assignments to MS/MS spectra and the number of resulting distinct peptide identifications. This can be explained by the fact that many peptides were sequenced multiple times, with some of the most abundant peptides sequenced more than 1,000 times (for example, peptides PAp00004784, PAp00003568, PAp00026910). While many correct peptide assignments to MS/MS spectra represent the same peptide sequence, the majority of incorrect peptide assignments are expected to be single identifications. As a result, the false-positive error rate on the level of distinct peptides is higher than that on the level of peptide assignments to MS/MS spectra.

Second, it should also be taken into account that a considerable fraction of all distinct peptides did not match any Ensembl entry. This is due to the fact that MS/MS spectra were searched against larger databases, such as human IPI, which contained a number of protein sequences not present in Ensembl. The fraction of all distinct peptide identifications that did not map to any Ensembl entry can be estimated using information provided in Table 2. Among peptides with probability of being correct of 0.99 or greater, only 2.6% of all distinct peptides did not map to any Ensembl sequence. The fraction of unmapped distinct peptides increases to 8.3% among peptides in the 0.95-0.99 probability range, 12.9% in the 0.9-0.95 range and 18.2% in the 0.7-0.9 range, reflecting the increase in the number of incorrect peptide identifications among peptides with lower probabilities. Thus, one can estimate that at least 18.2% of all incorrectly identified peptides did not map to any entry in Ensembl.

The false-positive error rate among distinct peptides that mapped to Ensembl (peptides with protein sequence match) can then be estimated to be not higher than the maximum possible number of distinct incorrect peptide identifications that have protein sequence matches (computed by multiplying the total number of peptide assignments to MS/MS spectra by the corresponding false-positive error rate and applying an 18.2% correction to account for peptides with no mapping to Ensembl) divided by the total number of peptides with protein sequence matches. The corresponding estimates are 16%, 6%, 3% and 0.8% in the case of minimum-probability thresholds 0.7, 9, 0.95 and 0.99, respectively (Table 2). It should be noted that these are conservative (upper bound) estimates and the actual error rate may be significantly smaller.

### Population of the database

The PeptideAtlas pipeline begins with the download of the Ensembl human protein database from [35]. Release 22.34d.1 (2004-06-02) was used here. PeptideAtlas peptides were then searched against the human proteins using BLAST with the following parameters adapted for searching small peptides [31]: -E 1 -W 2 -M PAM30 -G 9 -e 10 -K 50 -b 50 -F F. The BLAST results were then filtered for identical matches and mapped into chromosomal coordinates using Bio::EnsEMBL and Bioperl [36] Perl modules. The results are uploaded into the PeptideAtlas database and then the Ensembl genome browser. The PeptideAtlas database can handle different PeptideAtlas builds, different organisms and different versions of underlying genome data for maximum flexibility.

## Figures and Tables

**Table 1:** Summary of PeptideAtlas results

	Human	*Drosophila*
Ensembl version	22.34d.1 2004-06-02	19.3a.2, 2003-07-01
Ensembl gene predictions	23758	13525 from Release 3.1 FlyBase
Ensembl gene transcripts	34091	18289
PeptideAtlas version	FullHumanEns22APD0704P0.9	Fly 2
PeptideAtlas peptides	26840	4406
Number of experiments	52	3
PeptideProphet probability threshold	0.9	0.9
PeptideAtlas mapped peptides	25754	4406
PeptideAtlas mapped proteins	9747	3107
PeptideAtlas mapped genes	6423	1876
Percentage of the genome	27 %	14 %

**Table 2:** Comparison of different probability thresholds that were applied to the MS results

Probability	≥ 0.70	≥ 0.90	≥ 0.95	≥ 0.99
Total number of passing spectra	245724	224793	211674	179410
Peptides	31290	26840	25022	21598
Distinct peptides with protein sequence matches	29393	25754	24172	21030
Number of mapped proteins	11612	9747	9016	8134
Number of simple reduced proteins	7097	5826	5383	4845
False-positive estimate MS/MS spectra	2.4%	0.9%	0.05%	0.01%
False-positive estimate with protein sequence matches	<16%	<6%	<3%	<0.8%
